# Two Origins for the Gene Encoding α-Isopropylmalate Synthase in Fungi

**DOI:** 10.1371/journal.pone.0011605

**Published:** 2010-07-15

**Authors:** Erica M. Larson, Alexander Idnurm

**Affiliations:** Division of Cell Biology and Biophysics, School of Biological Sciences, University of Missouri-Kansas City, Kansas City, Missouri, United States of America; University College Dublin, Ireland

## Abstract

**Background:**

The biosynthesis of leucine is a biochemical pathway common to prokaryotes, plants and fungi, but absent from humans and animals. The pathway is a proposed target for antimicrobial therapy.

**Methodology/Principal Findings:**

Here we identified the *leuA* gene encoding α-isopropylmalate synthase in the zygomycete fungus *Phycomyces blakesleeanus* using a genetic mapping approach with crosses between wild type and leucine auxotrophic strains. To confirm the function of the gene, *Phycomyces leuA* was used to complement the auxotrophic phenotype exhibited by mutation of the *leu3^+^* gene of the ascomycete fungus *Schizosaccharomyces pombe*. Phylogenetic analysis revealed that the *leuA* gene in *Phycomyces*, other zygomycetes, and the chytrids is more closely related to homologs in plants and photosynthetic bacteria than ascomycetes or basidiomycetes, and suggests that the Dikarya have acquired the gene more recently.

**Conclusions/Significance:**

The identification of *leuA* in *Phycomyces* adds to the growing body of evidence that some primary metabolic pathways or parts of them have arisen multiple times during the evolution of fungi, probably through horizontal gene transfer events.

## Introduction

Many microbes are capable of growth under diverse conditions due to their ability to utilize whatever nutrients are available and produce *de novo* metabolites that are essential for life. This contrasts to animal species that rely on acquiring many basic metabolites, such as the essential amino acids, from food. The evolution of microbial metabolism is thus a fascinating area of investigation, particularly in the fungal kingdom. Why fungi can produce all 20 amino acids required for building proteins, whereas humans are unable to synthesize eight of these, can be explored through analysis of diverse animal and fungal species to assess whether this reflects gene loss in animals or gain in fungi, or a combination.

The origins of several biochemical pathways have derived from duplications of an ancestral enzyme or set of enzymes with broader substrate specificity than observed in current enzymes. One example is the divergence of related pathways for the biosynthesis of leucine, lysine, arginine and glutamate from a single ancestral pathway [1]. An Archaeon species *Pyrococcus horikoshii* encodes enzymes that retain dual-specificity for at least two of these amino acid biosynthetic pathways [2], [3]. In the case of leucine, its biosynthesis is of practical interest for a number of reasons. First, the pathway (Figure S1) is absent from animals, thus representing a possible target for antibiotic therapy. Furthermore, leucine biosynthesis is required for full virulence in a fungal model of disease using a pathogenic isolate of *Saccharomyces cerevisiae* [4]. It also contributes to virulence in bacterial pathogens of animals such as *Mycobacterium tuberculosis*, *M. bovis*, *Salmonella enterica* serovar Typhi and *Brucella suis*, and the plant pathogen *Xanthomonas oryzae* [5], [6], [7], [8], [9]. Second, the enzymes and related proteins are required for the biosynthesis of both leucine and glucosinolates in certain Brassicales plant species, such as *Arabidopsis thaliana* in which they have been studied [10], [11]. Third, the pathway has been investigated as a model system to understand regulatory control mechanisms. For instance, in the yeast *S. cerevisiae* there is a complex system of control in which at least four inputs regulate pathway output. This includes allosteric inhibition by leucine on the first enzyme of the pathway, α-isopropylmalate synthase (α-IPMS), to control its enzymatic activity [12], [13]. Similar allosteric inhibition of α-IPMS also occurs in multiple bacterial species.

*Phycomyces blakesleeanus* is a zygomycete fungus classified in the subphylum Mucormycotina, and is well studied because of its environmental sensing abilities, responses, and its ability to synthesize the pigment β-carotene [14]. It is a valuable organism from an evolutionary standpoint because the zygomycetes diverged early in the evolution of the fungal kingdom [15]. Thus, it represents a key lineage that can provide insight when compared to other later diverging fungi such as the ascomycetes and basidiomycetes. The origins of different metabolic pathways in the eukaryotes are relatively unexplored areas, in particular in fungi that represent the closest lineage to animals [15]. In this study we identify the gene encoding α-isopropylmalate synthase in *P. blakesleeanus* and show that fungi have two different origins for this enzyme.

## Materials and Methods

### Phycomyces blakesleeanus strains and genetic crosses

*P. blakesleeanus* strains used were NRRL1555 [wild type, *sex* (−)]; UBC21 [wild type, *sex* (+)], and A721 [*leuA purC sex* (−)], that is derived from the original *leu-51* H1 strain (see Figure S2 for the relationships between these strains). NRRL1555 is the most commonly used wild type strain and that used for the genome sequencing project. Strain UBC21 was sequenced using Solexa technology to identify polymorphisms with the NRRL1555 strain. Both genome sequences were generated by the US Department of Energy Joint Genome Institute. The double *leuA purC* mutant strain A721 was used to facilitate the phenotyping of the progeny from the A721 and UBC21 cross. Because *leuA* and *purC* are linked markers, it was clear that when the progeny was mutant for one phenotype but wild type for the other a genetic recombination event had occurred. This helped pinpoint the *leuA* mutation to a small part of the *P. blakesleeanus* genome and provided evidence for recombination in the progeny from the cross.

Crosses were established on V8 juice (5% solidified with 4% agar) medium supplemented with adenine and leucine (20 mg/L each). Zygospores were harvested and placed on wet filter paper. Two to three months later, these zygospores germinated and the progeny were isolated onto potato dextrose agar (Difco) supplemented with leucine and adenine. 116 progeny were isolated from individual zygospores, of which 104 were used for genetic mapping. Irregular progeny are common from *P. blakesleeanus* crosses [16]. The other 12 progeny were excluded because they appeared to be heterozygous for both alleles of the *sex* locus and/or exhibited reduced asexual sporulation compared to wild type, suggesting they are aneuploid.

Leucine and adenine auxotroph phenotypes were scored by plating asexual sporangiospores onto two different YNB media (0.67% yeast nitrogen base (Difco), 2% glucose): one supplemented with adenine and the other supplemented with leucine (20 mg/L). The plates were incubated at room temperature for 2–4 days and growth was observed to score the phenotypes. To induce a colonial growth morphology, media was acidified with 1 M HCl acid after autoclaving.

DNA extraction for fungal strains was performed on lyophilized mycelia disrupted with 2 mm diameter glass beads. Extraction buffer (1% CTAB, 1% β-mercaptoethanol, 10 mM EDTA, 0.7 M NaCl, 100 mM Tris-HCl, pH 7.5; 10 mL) was added and the samples incubated at 65°C for 30 min. After one chloroform extraction (10 mL), the DNA was precipitated in an equal volume of isopropanol. The DNA pellet was washed in 70% ethanol, dried and resuspended in 10 mM Tris-Cl, pH 8.5.

### Molecular markers and mapping

Polymorphic regions between strain A721 and UBC21 were identified from the *P. blakesleeanus* genome website (version 1.1; http://genome.jgi-psf.org/Phybl1/Phybl1.home.html), with those that could be detected by changes in restriction enzyme sites chosen to develop PCR-RFLP markers. The primers used to map the *leuA* gene in context to the surrounding DNA are listed in Table 1, along with other primers to amplify markers that were used to demonstrate independent assortment in the progeny. To assign alleles in each strain, the regions were amplified by PCR from genomic DNA, digested with restriction enzymes that distinguish each allele, and resolved on 0.8 or 1.0% agarose gels. In cases of ambiguous allele assignment, strains were re-isolated from 30% glycerol stocks stored at −80°C, DNA extracted, and the strains genotyped again. In situations where alleles could not be accurately assigned, these data were excluded from calculating segregation values.

**Table 1:** Oligonucleotide primers used in this study.

Name	Sequence (5′-3′)	Marker position/comments
Amplification of *leuA* from cDNA of *Phycomyces*.		
ALID0522	CATATGCCTGCCACTACTGAC	NdeI sites underlined.
ALID0523	CATATGTTAGACATCTACAGTGCG	
Molecular markers for PCR-RFLP mapping		
	Contig 15	
ALID0279	ATGCGCAGAAGACTTGAG	339,186 HindIII
ALID0280	TAAAATACCTGGTGCTGG	
ALID0524	TTGGCACTTTGACTGAGG	26,945 XhoI
ALID0525	TATCGCATTCTACTTGCC	
ALID0469	AGGAGAGATGGTTCTCTG	225,851 HpyCH4IV (*leuA*)
ALID0474	TAGCCAACAGTATCAGGG	
ALID0602	TCAAGTACAGAAATCAGC	919,915 EcoRV
ALID0603	AAGATTCAGCAAAGACAC	
EL007	GGAAAGTAACAGATGCAG	151,498 EcoRI
EL008	TCAGCAGTGATGTCTAAG	
EL009	CTTTGATGGCAGCAACAG	272,222 sequence SNP
EL010	GATGCCTGCAGATCCAAC	
	Contig 36	
ALID0247	TCTTGCTGGTTACTTCCG	140,027 BamHI (*purC*)
ALID0248	TCATCTTCATGGACATGG	
ALID0526	ATAAGGATAATCTCACCG	371,602 XbaI
ALID0527	TAGTTCAGGATTTCAGCC	
	Independent markers	
	Contig 8 (*pyrG*)	
ALID0251	AGTTGAACTTACTTACGC	956,230 EcoRV
ALID0252	TCTAGAACTAGAAGACTC	
	Contig 47 (*sex*)	
ALID0397	CCATTTGTAGGGTGAAG	143,449 XhoI
ALID0398	GCTAAATCAACAGAGTCC	
Amplification of *purC*		
EL011	CACTTGTCACTTGTCTTC	Forward 1
EL015	GATGCTGCCAGTCGTGTT	Forward 5
EL016	CTTGTGAGTGTTGACGAC	Reverse 1
EL019	TGTCATCGTTAGTAGGGC	Reverse 4
Disruption of *leu3* ^+^ in *S. pombe*		
ALID0504	TTGAATAGTCCTCAAACG	
ALID0505	GTCGACCTGCAGCGTACGTTCAATCAGATGACGAAC	KanMX F underlined
ALID0506	CGAGCTCGAATTCATCGACACTCTTTCTAGCAACGG	KanMX R underlined
ALID0507	AGATTACTTAGCAAATGC	
KanMX F	CGTACGCTGCAGGTCGAC	
KanMX R	TCGATGAATTCGAGCTCG	

### Phylogenetic analysis

Protein sequences of known or predicted α-isopropylmalate synthases were downloaded from GenBank, the Broad Institute (http://www.broadinstitute.org/science/projects/fungal-genome-initiative/fungal-genome-initiative) or the Joint Genome Institute (http://www.jgi.doe.gov/genome-projects/), based in part on species used to create a previous phylogeny of the protein (Figure 3A in reference [17]) and including characterized α-isopropylmalate synthases. Genes were identified in these databases using BLASTp and tBLASTn searches with the predicted amino acid sequences of the *Phycomyces leuA* or *S. cerevisiae LEU4* genes. ESTs were sought in the GenBank EST_others database, and the sequences translated into amino acids for inclusion in the phylogenetics analysis. Amino acids were aligned with ClustalW [18] and this initial alignment edited manually in MacClade 4.08 (included as supporting File S1) [19]. The alignment was analyzed in ProtTest v. 2.3 to determine the most appropriate protein model for subsequent analysis [20]. Maximum likelihood trees were generated using the PhyML (LG model) algorithm with SeaView v. 4 software [21]. To assess clade support, 100 bootstrap replicates were used in SeaView. The tree was exported from SeaView and reannotated in the Canvas 9 drawing program.

### Construction of a *Schizosaccharomyces pombe leu3*Δ deletion strain and its complementation with the *Phycomyces leuA* gene

**Figure 1:**
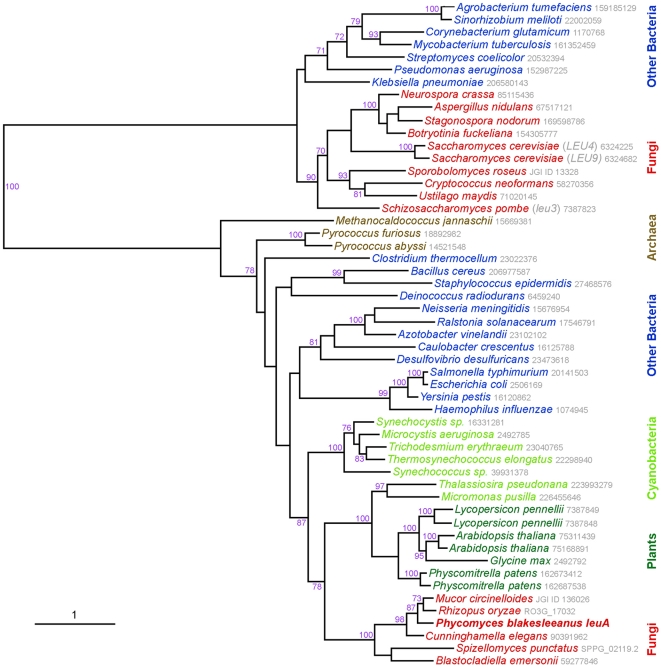
Maximum likelihood phylogenetic tree of known and putative α-isopropylmalate synthase proteins. Predicted amino acid sequences were downloaded from GenBank, with gi numbers provided after each species in grey, with the exception of *Phycomyces* which was sequenced during this study, and the *R. oryzae* and *S. punctatus* sequences from the Broad Institute, and *M. circinelloides* and *S. roseus* from the Joint Genome Institute. The amino acid sequences for *B. emersonii* and *C. elegans* are partial, being derived from expressed sequence tags. Bootstrap values (100 replicates) above 70% are shown.

*S. pombe* resources were provided by the Yeast Genetic Resource Center, Osaka, Japan. *S. pombe* strain L972 (YGRC reference FY7507: genotype *h* ^−^) was used as wild type and the uracil auxotroph strain MM72-4A (FY6843: genotype *ura4D18 h* ^−^) was used for gene replacement. The *leu3^+^* gene, predicted to encode α-isopropylmalate synthase (Figure 1), was targeted for deletion using a homologous recombination replacement strategy. A dominant selectable marker conferring G418 resistance (KanMX6) was amplified from plasmid pFA6-S65TGFP-KanMX6 with primers KanMX F and KanMX R. 5′ and 3′ flanks of *S. pombe leu3^+^* were amplified from wild type L972 genomic DNA using primers ALID0504 with ALID0505, and ALID0506 with ALID0507. The three PCR products were used in overlap PCR with primers ALID0504 and ALID0507. Primer sequences are provided in Table 1. The construct was transformed into strain MM72-4A using the lithium acetate-PEG method. Cells were recovered overnight in liquid media (0.5% yeast extract, 1% bacto-peptone, 3% glucose) at 30°C with shaking, plated onto medium containing G418 (50 µg/mL and 100 µg/mL), and grown at 30°C.

Gene replacement of *leu3* ^+^ was confirmed by PCR and Southern blot analysis. One *leu3*Δ strain, EML6, was used for subsequent analyses. For Southern blotting, 2 µg DNA was digested with restriction enzymes resolved on 0.8% agarose gels and blotted to Zeta-Probe nitrocellulose membranes (Bio-Rad, Hercules, CA) in 0.4 M NaOH. The blots were hybridized overnight with a probe comprised the *leu3^+^* gene and flanking regions, amplified with primers ALID0504 and ALID0507 from strain L972. The probe was labeled with [α-^32^P]-dCTP using the RediPrime II kit (Amersham GE Healthcase, Piscataway, NJ) according to manufacturer's recommendations.

The *leuA* open reading frame, without the introns, was amplified from wild type *P. blakesleeanus* cDNAs generated with Superscript III reverse transcriptase (Invitrogen, Carlsbad, CA) with primers ALID0522 and ALID0523, cloned into the pCR2.1 TOPO vector (Invitrogen) following manufacturer's suggestions, and plasmids sequenced. An insert was excised from one plasmid using NdeI restriction enzyme. *S. pombe* expression vector pTN157 (FYP424) was modified to remove an inserted gene with BamHI, and self-ligated to reconstitute the pREP42 vector. The *P. blakesleeanus leuA* gene was ligated into the NdeI site of the pREP42 plasmid, enabling constitutive expression from the “medium” strength *nmt* promoter [22]. This plasmid, pEML1, and empty control were transformed into the *S. pombe* wild type and *leu3*Δ deletion strains. Transformants were selected, based on the *ura4* ^+^ gene in the pREP42 backbone, on YNB media supplemented with leucine.

## Results

### *Phycomyces* contains a gene encoding a predicted α-isopropylmalate synthase that is unlike those found in Dikarya fungal species

*Phycomyces blakesleeanus* is a zygomycete fungus that is well studied for its abilities to sense and respond to light and to produce carotenoid pigments and their derivatives. In studies to generate a high-resolution genetic map for cloning genes required for light-sensing (Chaudhary and Idnurm, unpublished data), it was observed that markers on two separate contigs of the genome sequencing project were linked. One of these contigs (#36, *Phycomyces* genome release v. 1.1) contained the *madA* gene, which encodes a predicted blue-light photoreceptor for phototropism [23]. Previous genetic mapping had found that *madA* was within a genetic linkage group comprising *ribC* (a riboflavin auxotroph), the centromere, *leuA* (a leucine auxotroph), and *purC* (a purine auxotroph) [24], [25]. A candidate for *purC* that would encode the enzyme phosphoribosylformylglycinamidine synthase in the *de novo* purine biosynthesis pathway is ∼20 kb from *madA* in the genome on contig 36. The second contig (#15) was examined for candidates for the *ribC* and *leuA* genes. Candidates for both genes were identified: *ribC* as a putative RNA pseudouridylate synthase and *leuA* as a putative α-isopropylmalate synthase (α-IPMS). α-isopropylmalate synthase catalyzes the first step in the biosynthesis of leucine from acetyl-CoA and α-ketoisovalerate (also known as 3-methyl-2-oxobutanoic acid). This is followed by two other enzymes specific to the pathway, α-isopropylmalate isomerase and β-isopropylmalate dehydrogenase, and a final broad-specificity transaminase (Figure S1). When the predicted α-IPMS protein was searched against the GenBank databases, the most similar matches were found to be non-fungal. This observation prompted an investigation into the origins and functions of this gene.

### Phylogenetic analysis supports two origins of α-isopropylmalate synthase in fungi

To assess the evolutionary relationship of the candidate *leuA* gene of *P. blakesleeanus*, phylogenetic analyses were used. Known and predicted α-isopropylmalate synthase sequences were downloaded from GenBank and genome sequencing centers, the sequences were aligned (File S1), and maximum likelihood was used to infer phylogenetic relationships. The *Phycomyes leuA* gene (and others from non-Dikarya fungi) groups in a clade distinct and distant from the Dikarya ascomycete and basidiomycete species (Figure 1). The phylogenetic analyses suggest that the *leuA* gene in *P. blakesleeanus* is most closely related to the gene from plants and photosynthetic bacteria. Bootstrap analysis is a method of resampling the original sequence data that provides a measure of reliability of phylogenetic trees. Bootstrap analysis with 100 resamplings on this data further supports these relationships (Figure 1) and the observation that there are two distinct types of gene encoding α-isopropylmalate synthase in the fungal kingdom.

Because *P. blakesleeanus* diverged early in the evolutionary history of the fungal kingdom, it is possible that it preserved the original form of the gene, whereas later-diverging species, such as the ascomycetes and basidiomycetes (the monophyletic Dikarya) have acquired the gene more recently. The alternative hypothesis to explain the phylogenetic data is that the *P. blakesleeanus* gene is derived from a recent horizontal gene transfer event from a plant or Cyanobacterium. The third hypothesis is that both genes have originated from independent horizontal gene transfer events. To examine these hypotheses, GenBank and fungal genome sequencing projects at the Broad Institute and Joint Genome Institute were examined for α-isopropylmalate synthase homologs. All examples of the known and putative α-isopropylmalate synthases in the ascomycete and basidiomycete genome projects are most closely related to each other (Figure 1, and data not shown). Genes that most closely match *P. blakesleeanus* were found in related Mucormycotina species, including in the recently completed genome sequence of *Rhizopus oryzae* [26] and the ongoing genome project for *Mucor circinelloides*. Among the expressed sequence tag (EST) database at GenBank, a representative was also found for the Mucormycotina species *Cunninghamella elegans*.

Other major lineages among the basal fungi are the chytrids. α-isopropylmalate synthase and the two other genes in the leucine biosynthetic pathway appear to be absent from the *Batrachochytrium dendrobatidis* (Chytridiomycota) genome, which is surprising given this species is pathogenic on amphibians in light of correlation between leucine auxotrophy and attenuated virulence in animal pathogens [4], [5], [6], [7], [8]. Genome projects for two other chytrids *Allomyces macrogynus* (Blastocladiomycota) and *Spizellomyces punctatus* (Chytridiomycota) are ongoing at the Broad Institute. *A. macrogynus* encodes multiple copies of the gene, whereas *S. punctatus* has a single copy: all appear more closely related to the *P. blakesleeanus* LEUA protein than the Dikarya fungal homologs (Figure 1, and data not shown). Amongst the GenBank ESTs, there is an α-IPMS representative from *Blastocladiella emersonii* [27]. The presence of genes most closely related to one another in the chytrids and zygomycetes, which are unlikely to be monophyletic, supports the hypothesis that this form of α-isopropylmalate synthase was ancesteral in the kingdom, and that the type found in the Dikarya is a more recently acquired gene. The *leuA* gene present in *P. blakesleeanus* and other basal fungi is potentially the product of another horizontal gene transfer event from a photosynthetic species.

As an additional investigation into the origins of these genes in eukaryotic organisms, the intron positions were examined in the eukaryotic homologs (Figure S3). Intron positions are often conserved across eukaryotic lineages [28]. For the α-IPMS homologs, lineage-specific intron positions were also observed. For instance, the ascomycetes and basidiomycete homologs could be distinguished on introns unique to each subphylum. The chytrids and zygomycetes shared introns, further implicating the gene as having a common origin in these two paraphyletic lineages. Examination of plant homologs revealed a set of 11 introns conserved in a lycophyte and monocotyledonous and dicotyledenous plants. None of these 11 positions was conserved with any fungal homologs (Figure S3). This analysis, therefore, cannot resolve whether or not land plants and fungi share a common origin for their α-IPMS homologs.

The enzymes encoding the second and third steps specific to the leucine biosynthesis pathway (Figure S1) from *P. blakesleeanus* were analyzed by BLAST to assess whether just the first gene or the whole pathway shares a common evolutionary history. Both the α-isopropylmalate isomerase and β-isopropylmalate dehydrogenase enzymes appear most closely related to other fungal homologs. This suggests that only the first enzyme in the pathway has the unusual distribution in the fungal kingdom.

While *P. blakesleeanus* is a harmless organism, some of its close relatives can cause diseases in humans and animals that can be particularly difficult to treat [29], [30]. α-isopropylmalate synthase has been proposed as a potential target to develop antibacterial drugs [31], [32]. The LeuA protein of *M. tuberculosis* has been crystallized and its structure resolved [31]. A drug developed based on a rational design from this structure may well be of broad specificity against this pathogen and the common human pathogenic fungi found in the Dikarya clade. However, the divergence between enzymes would warrant caution in testing such a molecule for treatment of mucormycoses.

### The *leuA* mutant strain of *Phycomyces* bears a point mutation in the gene predicted to encode α-isopropylmalate synthase

**Figure 2:**
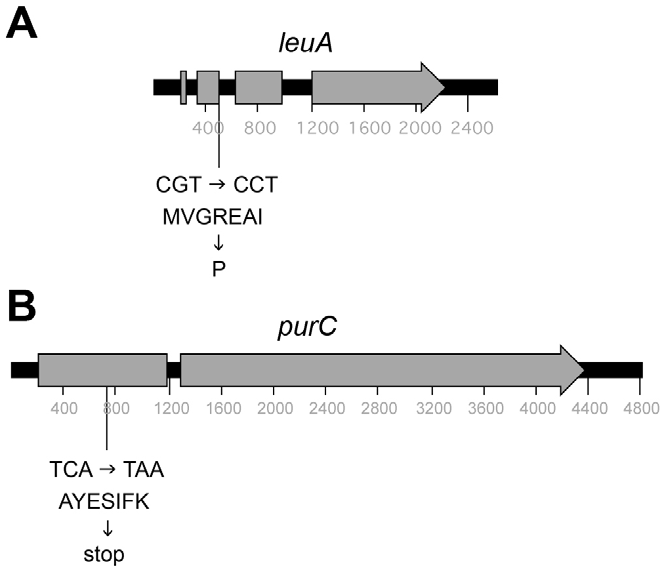
Position and effects on amino acid sequence of the point mutations in the *leuA* and *purC* genes of *Phycomyces* strain A721. Exons are drawn as grey boxes. The affected codons for the amino acid sequence within the surrounding context are provided, resulting in an R75P substitution in *leuA* mutants and S174* substitution in *purC* mutants.

The original *leuA* mutant strain, H1, was isolated during the 1973 Cold Spring Harbor *Phycomyces* course organized by Max Delbrück. It was used in the first genetic studies demonstrating meiosis in this fungus [33], [34], but the nature of the mutation has remained unknown and only a single mutant allele has been isolated. Preliminary genetic mapping based around the genome project and the phylogenetic analysis described above suggested that the gene on sequencing contig 15 could correspond to *leuA*. This gene was amplified by PCR and sequenced from strain A721, which bears the original *leu-51* mutant allele. A single base pair G-C substitution was observed in the A721 sequence compared to that of the wild type strain at the genome sequencing project. The gene was amplified from the wild type NRRL1555 strain and sequenced to ensure the bp substitution of A721 was not present in the wild type isolate (gene sequence submitted to GenBank as accession GQ465942). The substitution will change an arginine residue to a proline (R75P) in the second exon (Figure 2). This amino acid lies in a region of low sequence similarity, so it is not immediately apparent whether or not this would affect the protein function.

A gene also named *leuA* has been characterized from the related Mucormycotina species *Mucor circinelloides* and is a commonly used selectable marker for transformation in this species [35]. However, in this species *leuA* encodes the putative α-isopropylmalate isomerase enzyme, rather than α-isopropylmalate synthase. The homologous *P. blakesleeanus* gene was cloned and named *leu1* and is able to complement the *M. circinelloides leuA* mutants [36], [37]. Because the *leuA* gene was named and reported in *P. blakesleeanus* prior to *M. circinelloides*, the name should be maintained.

The *leuA* gene is linked to both *madA* and *purC* in *P. blakesleeanus* [24], [25]. Analysis of genes close to *madA* on contig 36 that could correspond to *purC* identified a gene predicted to encode phosphoribosylformylglycinamidine synthase, which is essential for *de novo* purine nucleotide biosynthesis in other organisms. This gene was amplified from strain A721 and sequenced. A single bp C-A substitution was observed in the sequence when compared to that of the wild type NRRL1555. This substitution changes a serine residue to a stop codon (S174*), which would truncate the protein (Figure 2). This finding is thus consistent with the previous genetic linkage data.

### Genetic segregation analysis links the candidate *leuA* gene mutation to the leucine auxotroph phenotype

**Figure 3:**
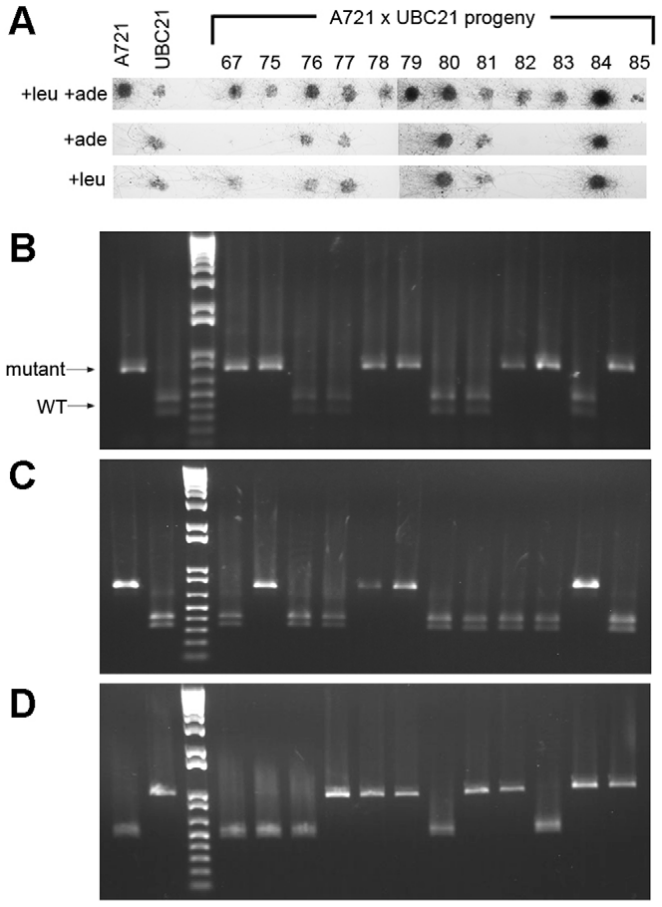
The *leuA* point mutation segregates with leucine auxotrophy. (A) Phenotypic analysis of A721, UBC21 and a subset of 12 of the 104 progeny from the A721 x UBC21 cross. Spores were plated onto YNB media containing HCl at a concentration of 5 mM, supplemented with leucine (+leu), adenine (+ade), or both (+leu +ade) to test for *leuA* and *purC* mutations. Growth on neither the YNB+leu nor the YNB+ade medium indicates that the strain is mutant for both the *leuA* and *purC* phenotypes, while growth on both indicates that the strain is WT. Growth on the YNB+ade medium with no growth on the YNB+leu medium indicates that the strain is an adenine auxotroph, mutant for the *purC* phenotype but WT for the *leuA* phenotype. (B) PCR of the *leuA* gene with primers ALID0469 and ALID0474, and digestion with HpyCH4IV (the G-C point mutation of A721 alters the ACGT site recognized by the HpyCH4IV restriction enzyme). (C and D) PCR of markers on two independent chromosomes confirm independent segregation. (C) PCR from the chromosome containing *pyrG* with primers ALID0251-ALID0252 and digestion with EcoRV. (D) PCR from the chromosome containing *sex* with ALID0397-ALID0398 and digestion with XhoI. DNA fragments were resolved on 1% agarose gels. The size markers between samples from strains A721 and UBC21 are the 1 kb + ladder (Invitrogen).

Successful stable transformation of DNA molecules into *P. blakesleeanus* has been an elusive method [38], and so Mendelian genetic analysis was used to confirm that the bp change identified in strain A721 confers the leucine auxotrophy. Strain A721 was crossed with a wild type strain of opposite sex type (UBC21), and 104 progeny isolated and scored for their ability to grow in the absence of leucine and adenine. Genomic DNA was extracted from these strains, and the putative *leuA* gene amplified with primers ALID0469 and ALID0474. The G-C bp difference between strains A721 and UBC21 alters the ACGT site recognized by the HpyCH4IV restriction enzyme, enabling a PCR-RFLP assessment of the two alleles at this locus (Figure 3). Out of the A721 x UBC21 progeny, 43 were assessed as wild type and all 43 bore the wild type *leuA* allele, while 61 were assessed as leucine auxotrophs and carried the *leuA* mutant allele.

To ensure that the progeny set used represented a recombinant population, markers unlinked to the chromosome on which *leuA* is located were sought. Previous genetic studies had used tetrad analysis to map centromeres, hence enabling specific chromosomes to be identified for *P. blakesleeanus* [25]. Strain A721 contains a part of the UBC21 genome (Figure S2), making the design of these markers a challenge. For instance, we found that markers linked to the chromosomes containing the previously identified *madB* or *carRA* genes [39], [40] were unsuitable for genetic analysis in this cross because the regions were identical between strain A721 and UBC21. However, the *sex* locus and *pyrG* gene are on separate chromosomes from *leuA* [41], [42], [43], and markers were developed successfully for these two regions. The frequency of recombination was analyzed between *leuA* and these two markers to test for independent assortment in the progeny set. As outlined in Table 2, progeny representing each of the eight expected genetic classes were obtained in roughly equal proportions, and these two markers segregated independently of *leuA* and each other. A χ^2^ test on these data showed that they were no different from the expected equal numbers of progeny due to chance (*P* = 0.62). For the *leuA* marker and the contig 47 *sex*-linked marker, the frequency of recombination was 52.0%. For the *leuA* marker and the contig 8 *pyrG*-linked marker, the frequency of recombination was 47.0%. This confirms that independent segregation has occurred during the cross to yield the progeny set in which the *leuA* mutant allele is linked to the leucine auxotrophy.

**Table 2:** Independent assortment of chromosomes in the progeny set from a cross between strains A721 (*leuA*) x UBC21 (wild type).

Marker	Allele							
*leuA*	A	U	A	U	U	A	U	A
Contig 47	A	U	U	A	A	A	U	U
Contig 8	A	U	U	A	U	U	A	A
No. progeny	14	12	17	8	11	11	17	12

Alleles from each strain are designed as A from A721 or U from UBC21, with representative progeny from each of the eight predicted classes. Markers on contigs 47 and 8 are on separate chromosomes to the *leuA* gene and each other.

The mutation in *purC* does not alter a restriction enzyme site so could not be assessed via PCR-RFLP. However, polymorphisms exist for the putative *purC* alleles between the two parents used in this cross. Primer pair ALID0247 and ALID0248 was used to amplify part of the *purC* gene that included a BamHI cut site in one but not the other allele. The A721 *purC* allele segregates with the purine auxotrophy, and UBC21 allele segregates with wild type growth in the absence of purine, supporting the hypothesis that this gene corresponds to *purC*.

**Figure 4:**
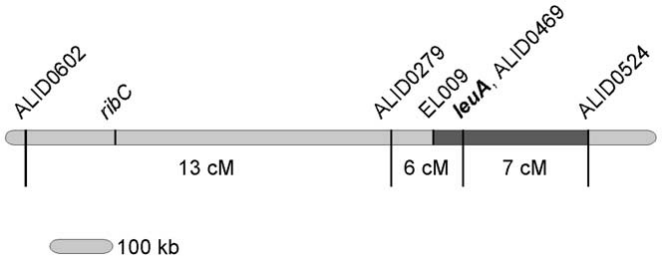
Diagram of the *Phycomyces* genome contig 15 illustrating positions of *leuA* and molecular markers and corresponding genetic distances. Recombination frequencies are based on segregation analysis from 104 progeny of a cross between strains A721 and UBC21. Marker names refer to one of the oligonucleotide primers used for amplification of the regions as PCR-RFLPs, as detailed in Table 1. Leucine auxotrophy maps to within the dark grey box. The location of a candidate for the *ribC* gene is marked.

Recombination was further characterized with respect to *purC* and markers on contig 15 for the 104 progeny of the A721 x UBC21 cross. The frequency of genetic recombination between *leuA* and *purC* was 9.6% (10 recombinants out of 104 progeny). This is consistent with previous recombination frequencies that ranged from 14-18% based on pooled random spore or tetrad analyses of crosses between near isogenic strains [24], [25]. Additional PCR primers were designed on contig 15 and alleles assigned for the 104 progeny to generate a map that is illustrated in Figure 4. Markers were designed closer to the *leuA* gene and used to examine a subset of the progeny. Two recombinant progeny that had undergone a crossing over event between the gene and marker were found on one side of *leuA* (but not the other side) that enabled the gene to be placed within a 245 kb interval region. This region includes 67 predicted genes, none of which are candidates to be involved in leucine biosynthesis with the exception of the gene we designate *leuA*.

### The *Phycomyces leuA* gene can complement a leucine auxotroph phenotype of a mutation in the ascomycete species *Schizosaccharomyces pombe*

**Figure 5:**
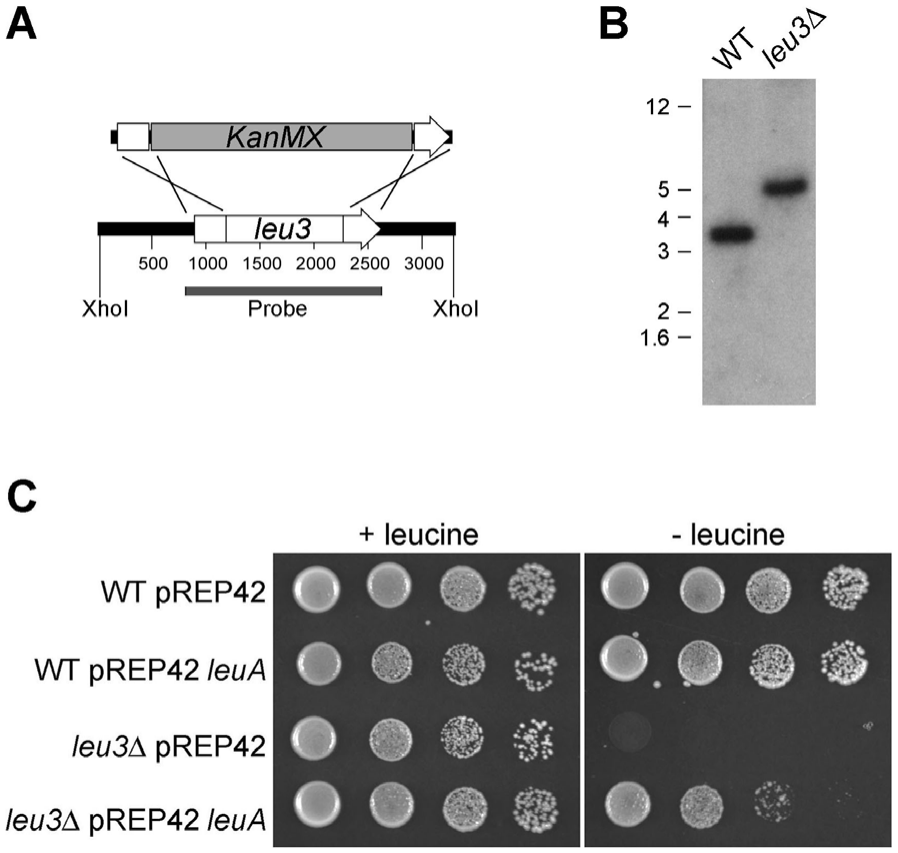
Deletion of the *S. pombe leu3* gene and complementation of the mutation with the *Phycomyces leuA* gene. (A) Diagram of the strategy to replace *leu3^+^* with the *KanMX* construct, position of XhoI restriction enzyme sites adjacent to the *leu3^+^* gene and absent from *KanMX*, and the DNA fragment used in Southern blot analysis. The white boxes on either side of *KanMX* represent 300 bp of homologous sequence to the *leu3^+^* gene. (B) Southern blot of genomic DNA of wild type or *leu3*Δ mutant strains digested with XhoI and hybridized with the wild type copy of *leu3^+^*. The sizes of molecular markers in kb are indicated adjacent to the autoradiograph. (C) 10-fold serial dilutions of *S. pombe* strains on YNB media supplemented with or without leucine. Photographs were taken after 4 days of growth at 30°C. Wild type (WT) or *leu3* deletion strains (*leu3*Δ) were transformed with empty plasmid (pREP42) or plasmid expressing the *P. blakesleeanus leuA* cDNA (pREP42 *leuA*).

As a final piece of evidence that the *leuA* gene of *P. blakesleeanus* encodes an α-IPMS enzyme, the *P. blakesleeanus* gene was used to complement the equivalent gene mutation in the ascomycete *S. pombe*. The *leu3^+^* gene in this species was targeted for gene replacement with the *KanMX6* marker, with 300 bp of DNA sequence corresponding to the 5′ and 3′ ends of the *leu3^+^* gene used to target the construct to the native locus by homologous recombination. Gene replacement was confirmed by PCR and Southern blot analysis, and that a single copy of the construct had integrated into the genome (Figure 5A, B, and data not shown). Of six deletion strains tested, all were unable to grow on medium that lacked leucine, implicating this gene as functioning in the leucine biosynthesis pathway of *S. pombe*. The cDNA of *P. blakesleeanus leuA* was amplified and cloned into a plasmid vector that enables expression of genes in *S. pombe* strains. The plasmid and empty vector control plasmid were transformed into one of the *S. pombe leu3*Δ deletion mutants and the wild type strain. The phenotypes of these *S. pombe* strains indicate that the *leu3*Δ mutant cannot grow without leucine added to the media, whereas transformation with the plasmid containing the *P. blakesleeanus leuA* gene partially rescues this phenotype (Figure 5C). The partial complementation may be due to a number of factors, including expression level from the *S. pombe nmt* promoter or divergence in protein properties.

## Discussion

Microbes are capable of diverse metabolic processes that are important for their survival and also directly impact human affairs. Most fungi synthesize *de novo* all 20 amino acids. In contrast animals such as ourselves do not, leading to the potential to target amino acid metabolism for antifungal (or antibacterial) drug design. It is unclear whether animals have lost or fungi have gained the ability to synthesize these amino acids. Here we investigate the origins of one enzyme found in fungi, but not humans, that encodes the first reaction in the biosynthesis of leucine, and show that there are two distinct families of enzymes in fungi that mediate this activity.

Many evolutionary studies, particularly with the increasing number of available genome sequences, use DNA sequence information solely to infer similar enzymatic or other function. Metabolic pathways may have common origins with overlapping substrate specificity, and so caution should be used in interpreting functional data based solely on DNA sequence analysis. Here we provide four pieces of evidence that the gene we name *leuA* of *P. blakesleeanus* encodes an α-IPMS, and that this gene has a distinct origin within the fungal kingdom. First, the *P. blakesleeanus leuA* gene clusters in phylogenetic analyses with homologous enzymes, including the *Arabidopsis thaliana* homologs that have been shown to be biochemically active [10]. In contrast, the Dikarya fungal homologs cluster in a distinct clade. Second, there is a point mutation in the *leuA* gene in a leucine auxotrophic strain of *P. blakesleeanus*. Third, this mutation co-segregates in genetic crosses between the wild type and mutant strain, and is linked to previously and newly identified genetic markers, but is unlinked to markers on independent chromosomes. Fourth, the *P. blakesleeanus leuA* gene can functionally complement a mutation in the putative α-IPMS gene of an ascomycete fungus.

This research suggests that the majority of the fungi, which are within the Dikarya clade, have acquired an alternative form of the α-IPMS gene. When and why this has occurred remains a mystery that may be resolved by further studies of the enzymatic properties of these proteins and how they are regulated. With recent increases in genome sequencing from diverse organisms, there is evidence for horizontal gene transfer (HGT) events being common in different fungal species and having occurred from multiple sources such as plants, other fungi, and prokaryotes [44], [45], [46], [47]. These events may account for small or large increases in numbers of genes [48], [49], [50]. It is striking that the *Phycomyces* form of α-IPMS clusters with others found in photosynthetic organisms, perhaps suggesting that there were two horizontal gene transfer events for this gene during evolution of the fungal kingdom. Examination of genome sequencing projects reveals that this form is also seen in diatoms, the coccolith *Emiliania huxleyi*, *Phytophthora* species and slime mold *Physarum polycephalum*, which would suggest that the acquisition of this gene was early in eukaryote evolution probably from a Cyanobacterium ancestor.

The hypothesis that α-IPMS genes were acquired in fungi through horizontal gene transfer events contributes to the growing body of research suggesting the widespread occurrence of HGT as a significant method of diversification for fungal genomes. Studies on various biosynthetic pathways in fungi propose that HGT has played an important role in the evolution of fungal genomes, and more generally in metabolic adaption in eukaryotes [44], [45]. For example, there is evidence that glycosyl hydrolases in rumen fungi are genetically similar to the same cellulose-degrading enzymes in bacteria, potentially the result of an HGT event facilitated by the sharing of a common environment [45]. Another study suggests that fungal 6-methylsalicylic acid synthase type polykediate synthase genes were acquired through HGT from bacterial sources, possibly by a Leotiomyceta ancestor that existed in a symbiotic relationship with bacteria [51]. The shikimate pathway present in ascomycete fungi represents another potential series of HGT events, as the enzymes are thought to originate from the plastid progenitor genome and to have been acquired in plants, prokaryotes, and ascomycete fungi through series of gene acquisitions and losses [52]. This transfer event parallels the events for the α-IPMS gene in the basal fungi. A previous analysis of α-IPMS homologs suggested that the targeting to of the enzyme to plastids reflected an origin from the endosymbiotic event giving rise to these organelles [17]. If so, an area of ambiguity is how the plastid-targeting information was modified during evolution such that the enzyme would function in the fungi. These several examples, as well as the potential HGT event that transferred α-IPMS genes to the fungi, demonstrate acquisition of biosynthetic pathway genes through HGT as a significant source of evolution and diversification for fungal genomes.

Understanding the factors that can regulate transcription of the *leuA* gene and thus change the rate of leucine biosynthesis can be important when considering the gene in any practical application, such as a target for antibiotic therapy or antifungal treatment [31], [53]. In some organisms, such as *S. cerevisiae*, feedback inhibition by leucine has been observed as a method of allosteric regulation of enzymatic activity [13]. However, α-isopropylmalate synthases are subject to many levels of control in both production and regulation of enzymatic activity. For example, another method of regulation in *S. cerevisiae* includes α-IPMS inactivation by CoA using a second binding site whose availability depends on the concentration of zinc and other divalent metal ions [13]. It has also been found in the ascomycete fungus *Neurospora crassa* that leucine feedback inhibits catalysis and represses synthesis of α-IPMS. In *N. crassa*, levels of α-IPMS can also dictate the production of α-isopropylmalate isomerase and β-isopropylmalate dehydrogenase, which catalyze the second and third steps of leucine biosynthesis respectively [54] (Figure S1). The structure of the α-IPMS enzyme in *Mycobacterium tuberculosis* has been solved and the enzyme shows a similar feedback inhibition mechanism [31]. The site of inhibition is at the C-terminal end of the protein, based on *S. cerevisiae* mutant strains that are no longer inhibited by leucine [55], [56] and the crystallization of leucine within the *M. tuberculosis* protein [31]. However, many of the key amino acids in this pocket or identified by mutant studies are not conserved in the *P. blakesleeanus* protein. Similarly, in the *A. thaliana IPMS1* and *IPMS2* genes these residues are diverged, and leucine provides only minimal feedback inhibition to heterologously-expressed or native enzymes [10]. While biochemical studies are required to assess if the *P. blakesleeanus* enzyme also shows feedback inhibition by leucine, it is possible that other regulatory mechanisms control the expression of this enzyme in this species. We tested, by Northern blots, levels of the *leuA* transcripts isolated from wild type *P. blakesleeanus* grown under a range of conditions, but did not observe any clear regulation in response to leucine or other amino acid concentrations in the media (data not shown).

The basal fungi are experiencing a resurgence in interest as their genome sequences become available, especially to understand early evolutionary events in the Opisthokont (ie. the animals and fungi) lineage. At present, a mere dozen or so protein-encoding genes have been characterized with corresponding mutant strains in the non-Dikarya lineages. The identification of the *P. blakesleeanus leuA* gene represents an example of the new evolutionary insights that may emerge about primary metabolism in the fungal kingdom from analysis of these species.

## Supporting Information

Figure S1 Leucine biosynthetic pathway.
(1.03 MB JPG)

Figure S2 Pedigree of the A721 strain of *Phycomyces blakesleeanus*. The genetic history of the original leucine auxotroph H1 (*leuA51*) and the crosses that gave rise to strain A721 are illustrated. Three induced mutagenic events occurred, using nitrosoguanidine (NG) or ultraviolet light (UV) as indicated with grey arrows.
(0.12 MB JPG)

Figure S3 Conservation of introns positions in α-isopropylmalate synthases within lineages. Intron positions, in the context of protein coding sequences, are colored if conserved in position between species, or in grey if unique to that species.
(0.86 MB JPG)

File S1 MacClade 4.08 alignment of α-isopropylmalate synthase proteins. This alignment was used to generate Figure 1.
(0.06 MB TXT)
