# Supplementary figures and images for: Two Origins for the Gene Encoding α-Isopropylmalate Synthase in Fungi
Source: PLoS One. 2010 Jul 15;5(7):e11605. doi: 10.1371/journal.pone.0011605 (PMC2904702; doi:10.1371/journal.pone.0011605)

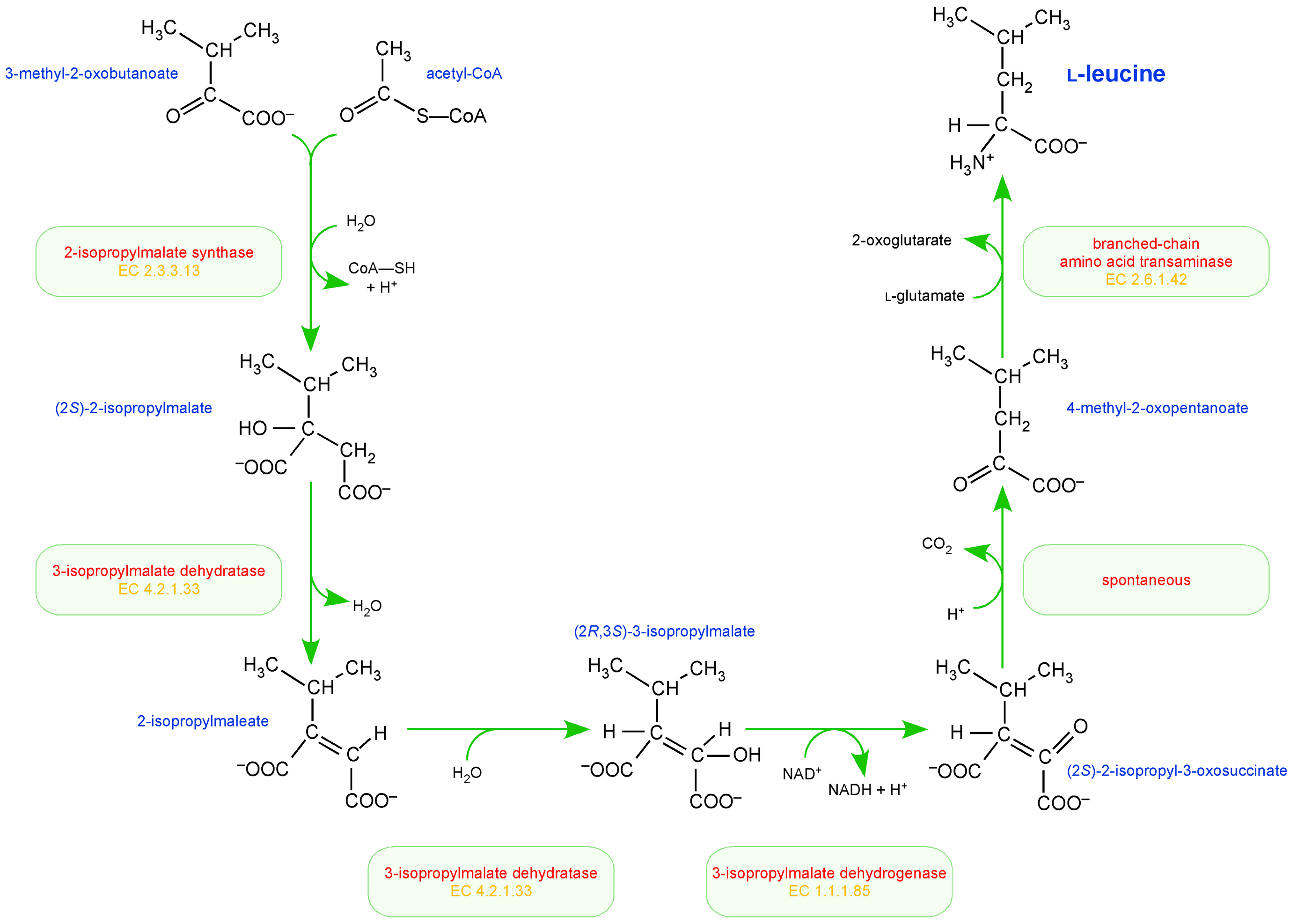

Supplement: Figure S1 — Leucine biosynthetic pathway. (1.03 MB JPG) [file pone.0011605.s001.jpg]

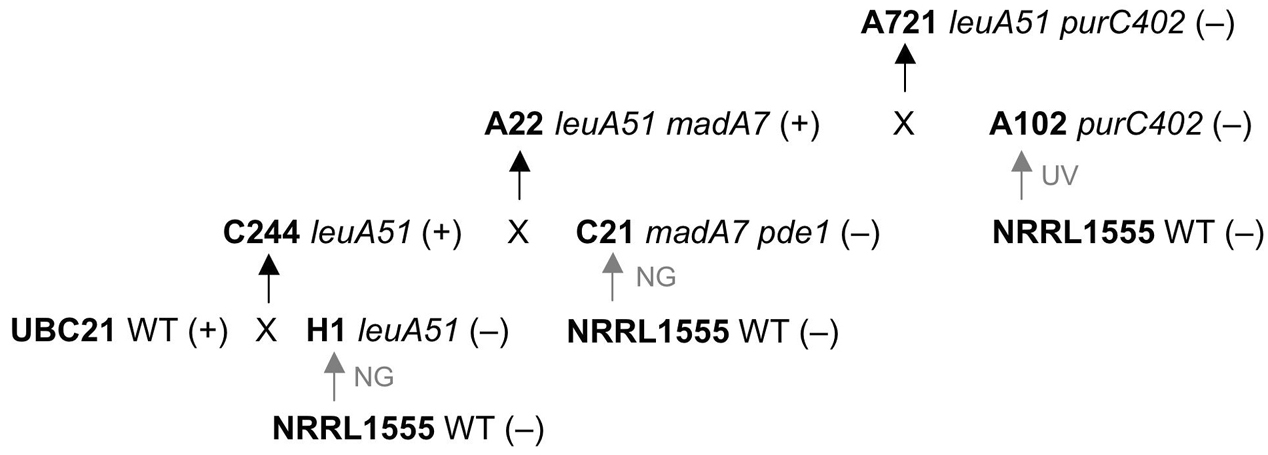

Supplement: Figure S2 — Pedigree of the A721 strain of Phycomyces blakesleeanus. The genetic history of the original leucine auxotroph H1 (leuA51) and the crosses that gave rise to strain A721 are illustrated. Three induced mutagenic events occurred, using nitrosoguanidine (NG) or ultraviolet light (UV) as indicated with grey arrows. (0.12 MB JPG) [file pone.0011605.s002.jpg]

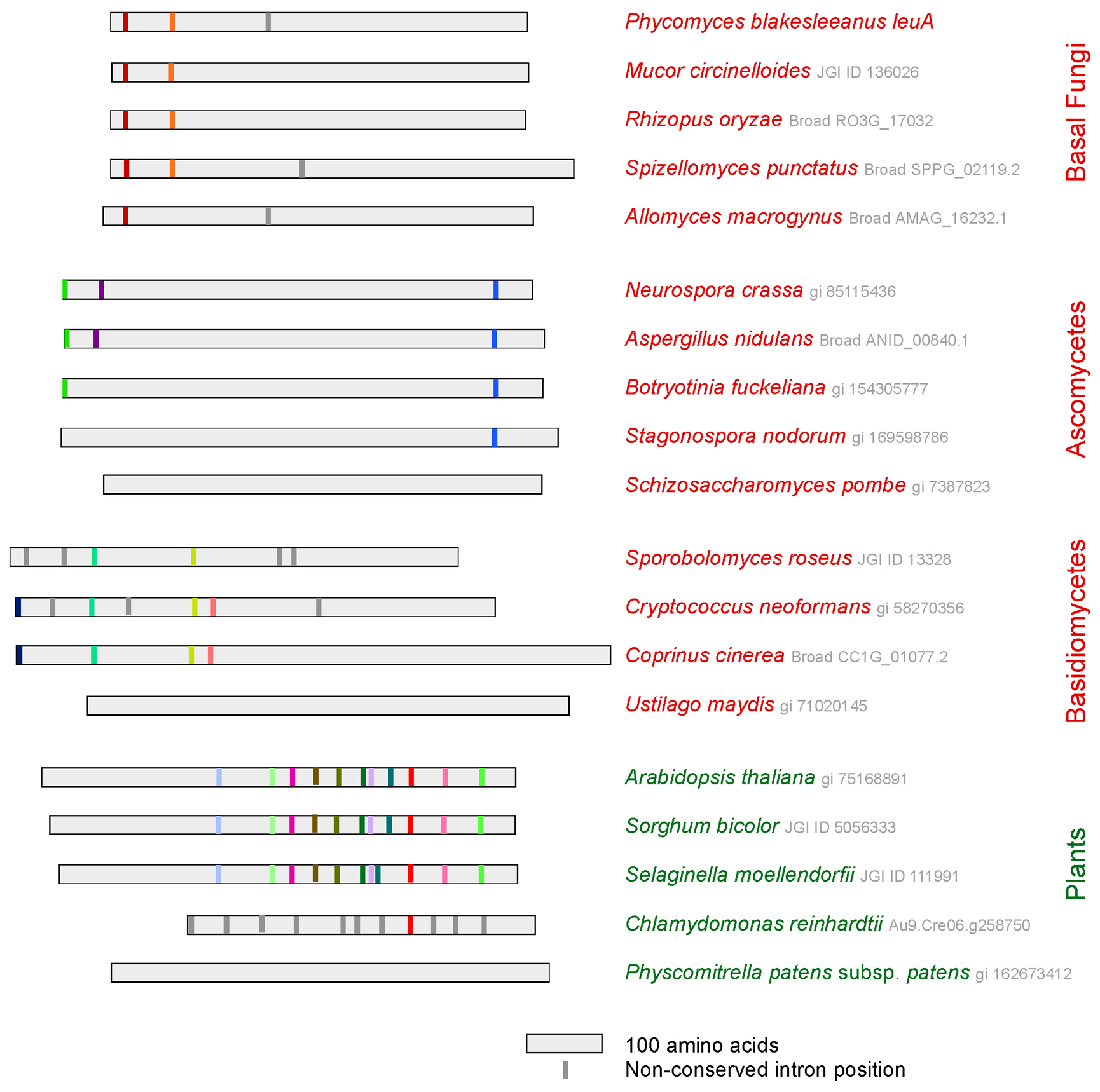

Supplement: Figure S3 — Conservation of introns positions in α-isopropylmalate synthases within lineages. Intron positions, in the context of protein coding sequences, are colored if conserved in position between species, or in grey if unique to that species. (0.86 MB JPG) [file pone.0011605.s003.jpg]
